# Propane-1,2-diaminium bis­(pyridine-2,6-dicarboxyl­ato-κ^3^ *O* ^2^,*N*,*O* ^6^)cadmate dihydrate

**DOI:** 10.1107/S160053681102438X

**Published:** 2011-06-25

**Authors:** Hossein Aghabozorg, Zeynab Khazaie, Ali Akbar Agah, Maryam Saemi, Behrouz Notash

**Affiliations:** a Faculty of Chemistry, Tarbiat Moallem University, 15614, Tehran, Iran; b Department of Chemistry, Shahid Beheshti University, G. C., Evin, Tehran, 1983963113, Iran

## Abstract

The reaction of cadmium nitrate dihydrate, propane-1,2-diamine and pyridine-2,6-dicarb­oxy­lic acid in a 1:1:2 molar ratio in an aqueous solution resulted in the formation of the title compound, (C_3_H_12_N_2_)[Cd(C_7_H_3_NO_4_)_2_]·2H_2_O or (*p*-1,2-daH_2_)-[Cd(pydc)_2_]·2H_2_O (where *p*-1,2-da is propane-1,2-diamine and pydcH_2_ is pyridine-2,6-dicarb­oxy­lic acid). The Cd^II^ ion is coordinated by four O and two N atoms of two pydc ligands in a distorted octa­hedral environment. The structure also contains two uncoordinated water mol­ecules. In the crystal, there are several inter­molecular N—H⋯O, O—H⋯O and weak C—H⋯O hydrogen bonds. There are also π–π stacking inter­actions between the pyridine rings of pydc units, with centroid–centroid distances of 3.4708 (16) Å.

## Related literature

For related proton-transfer compounds reported by our group, see: Aghabozorg *et al.* (2008*a* ▶,*b* ▶,*c* ▶,*d* ▶); Pasdar *et al.* (2011 ▶).
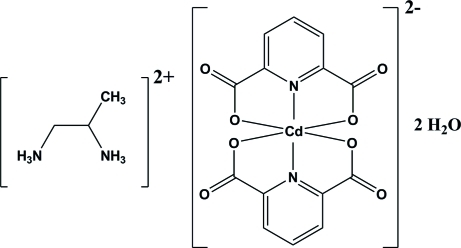


## Experimental

### 

#### Crystal data

(C_3_H_12_N_2_)[Cd(C_7_H_3_NO_4_)_2_]·2H_2_O
*M* *_r_* = 554.80
Triclinic, 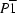

*a* = 8.6227 (17) Å
*b* = 10.133 (2) Å
*c* = 13.448 (3) Å
α = 81.36 (3)°
β = 76.73 (3)°
γ = 65.38 (3)°
*V* = 1037.7 (5) Å^3^
*Z* = 2
Mo *K*α radiation
μ = 1.12 mm^−1^
*T* = 298 K
0.30 × 0.20 × 0.15 mm

#### Data collection

Stoe IPDS 2T diffractometer
Absorption correction: numerical (*X-SHAPE* and *X-RED32*; Stoe & Cie, 2005 ▶)*T*_min_ = 0.764, *T*_max_ = 0.842
11690 measured reflections
5554 independent reflections
4562 reflections with *I* > 2σ(*I*)
*R*_int_ = 0.035

#### Refinement

*R*[*F* ^2^ > 2σ(*F* ^2^)] = 0.030
*wR*(*F* ^2^) = 0.063
*S* = 1.02
5554 reflections
330 parameters
1 restraint
H atoms treated by a mixture of independent and constrained refinement
Δρ_max_ = 0.50 e Å^−3^
Δρ_min_ = −0.37 e Å^−3^

### 

Data collection: *X-AREA* (Stoe & Cie, 2005 ▶); cell refinement: *X-AREA*; data reduction: *X-AREA*; program(s) used to solve structure: *SHELXS97* (Sheldrick, 2008 ▶); program(s) used to refine structure: *SHELXL97* (Sheldrick, 2008 ▶); molecular graphics: *ORTEP-3 for Windows* (Farrugia, 1997 ▶); software used to prepare material for publication: *WinGX* (Farrugia, 1999 ▶).

## Supplementary Material

Crystal structure: contains datablock(s) I, global. DOI: 10.1107/S160053681102438X/bt5545sup1.cif

Structure factors: contains datablock(s) I. DOI: 10.1107/S160053681102438X/bt5545Isup2.hkl

Additional supplementary materials:  crystallographic information; 3D view; checkCIF report

## Figures and Tables

**Table 1:** Hydrogen-bond geometry (Å, °)

*D*—H⋯*A*	*D*—H	H⋯*A*	*D*⋯*A*	*D*—H⋯*A*
N3—H3*A*⋯O10^i^	0.89 (3)	1.90 (3)	2.780 (3)	172 (3)
N3—H3*B*⋯O3^ii^	0.86 (3)	2.04 (3)	2.899 (3)	177 (3)
N3—H3*C*⋯O2^i^	0.92 (2)	1.89 (2)	2.790 (3)	164 (3)
N4—H4*A*⋯O8^ii^	0.91 (3)	1.96 (3)	2.870 (3)	176 (3)
N4—H4*B*⋯O5	0.83 (3)	2.06 (3)	2.889 (3)	172 (3)
N4—H4*C*⋯O9^ii^	0.91 (3)	1.90 (3)	2.803 (3)	170 (3)
O9—H9*A*⋯O4^iii^	0.76 (4)	1.96 (4)	2.708 (3)	170 (4)
O9—H9*B*⋯O2^iv^	0.84 (4)	2.00 (4)	2.827 (3)	165 (4)
O10—H10*A*⋯O6^v^	0.88 (6)	2.04 (6)	2.848 (4)	151 (6)
O10—H10*B*⋯O8^vi^	0.74 (4)	2.19 (4)	2.835 (3)	147 (4)
C10—H10⋯O3^vii^	0.93	2.54	3.298 (3)	139
C12—H12⋯O1^vi^	0.93	2.44	3.200 (3)	139

Symmetry codes: (i) 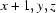; (ii) 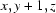; (iii) 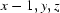; (iv) 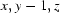; (v) 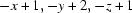; (vi) 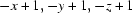; (vii) 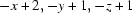.

**Fig. 1.**
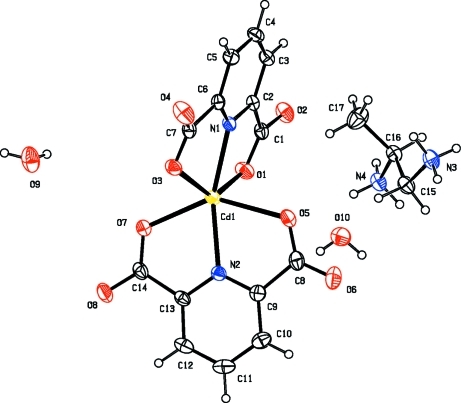
The molecular structure of the title compound with displacement ellipsoids drawn at 30% probability level.

**Fig. 2.**
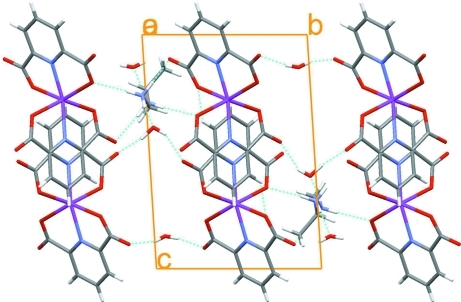
The packing diagram of the title compound viewed down the a-axis. The intermolecular N—H···O, O—H···O and weak C—H···O hydrogen bonds are shown as blue dashed lines.

**Fig. 3.**
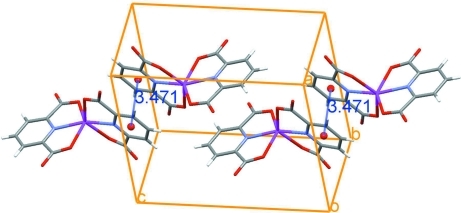
The packing diagram of the title compound showing π-π interactions between pydc fragments.Only anionic parts are shown for clarity.

## supplementary crystallographic information

### Comment

Our group has previously reported some proton transfer systems, using pyridine-2,6-dicarboxylic acid(pydcH_2_), propane-1,2-diamine(*p*-1,2-da) and propane-1,3-diamine (*p*-1,3-da) which formed the proton transfer compounds (pnH_2_)(pydcH)_2_.2H_2_O(Aghabozorg, *et al.*, 2008*d*), (*p*-1,3daH_2_)[Cd(pydc)_2_]. 3.5H_2_O (Aghabozorg, *et al.*, 2008*b*), (C_3_H_12_N_2_)[Ni(C_7_H_3_NO_4_)_2_].4H_2_O (Hossein Aghabozorg *et al.*, 2008*c*), (pnH_2_)[Hg(hypydc)Cl(H_2_O)]_2_.4H_2_O (Aghabozorg, *et al.*, 2008*a*) and (*p*-1,2-daH_2_)[Zr(pydc)_3_].3H_2_O(Pasdar, *et al.*, 2011).

The crystal structure of the title compound is shown in Fig. 1. The Cd(II) ion is six coordinated by two (pydc)^2-^ groups in a distorted octahedral environment. The torsion angles O(5)—Cd(1)—O(3)—C(7) and O(3)—Cd(1)—O(5)—C(8) are 67.86 (15)° and 108.19 (17)° respectively, and also the angles O(7)—Cd(1)—O(1) and O(3)—Cd(1)—O(5) are 103.92 (7)° and 105.44 (6)° respectively, indicate that the two (pydc)^2-^ units are not  perpendicular to one another. In the crystal structure of the title compound, there are several intermolecular N—H···O, O—H···O and weak C—H···O hydrogen bonding (Table 1 & Fig. 2). There are also π–π stacking interactions between the pyridine rings of pydc moieties with a centroid to centroid distance of 3.4708 (16) Å (Fig. 3).

### Experimental

An aqueous solution (30 ml) of propane-1,2-diamine(1 mmol), pyridine-2,6-dicarboxylic acid (2 mmol) and cadmium(II) nitrate dihydrate(1 mmol) were stirred at room temperature. Colorless crystals of the title compound were obtained after three weeks at room temperature.

### Refinement

H atoms attached to O and N were found in a difference Fourier map and refined isotropically. H3C were refined with distance restraints of N—H 0.92 (2). Other H atoms were positioned geometrically and refined as riding atoms with C—H = 0.93–0.98 Å, *U*iso(H) = 1.5*U*eq(C) for methyl H atoms and 1.2*Ueq*(C) for the others.

### Crystal data

**Table:**

(C_3_H_12_N_2_)[Cd(C_7_H_3_NO_4_)_2_]·2H_2_O	*Z* = 2
*M**_r_* = 554.80	*F*(000) = 560
Triclinic, *P*1	*D*_x_ = 1.776 Mg m^−^^3^
Hall symbol: -P 1	Mo *K*α radiation, λ = 0.71073 Å
*a* = 8.6227 (17) Å	Cell parameters from 5554 reflections
*b* = 10.133 (2) Å	θ = 2.2–29.2°
*c* = 13.448 (3) Å	µ = 1.12 mm^−^^1^
α = 81.36 (3)°	*T* = 298 K
β = 76.73 (3)°	Plate, colorless
γ = 65.38 (3)°	0.3 × 0.2 × 0.15 mm
*V* = 1037.7 (5) Å^3^	

### Data collection

**Table:**

Stoe IPDS 2T diffractometer	5554 independent reflections
Radiation source: fine-focus sealed tube	4562 reflections with *I* > 2σ(*I*)
graphite	*R*_int_ = 0.035
Detector resolution: 0.15 mm pixels mm^-1^	θ_max_ = 29.2°, θ_min_ = 2.2°
rotation method scans	*h* = −10→11
Absorption correction: numerical (*X-SHAPE* and *X-RED32*; Stoe & Cie, 2005)	*k* = −13→13
*T*_min_ = 0.764, *T*_max_ = 0.842	*l* = −18→18
11690 measured reflections	

### Refinement

**Table:**

Refinement on *F*^2^	Primary atom site location: structure-invariant direct methods
Least-squares matrix: full	Secondary atom site location: difference Fourier map
*R*[*F*^2^ > 2σ(*F*^2^)] = 0.030	Hydrogen site location: inferred from neighbouring sites
*wR*(*F*^2^) = 0.063	H atoms treated by a mixture of independent and constrained refinement
*S* = 1.02	*w* = 1/[σ^2^(*F*_o_^2^) + (0.030*P*)^2^ + 0.1592*P*] where *P* = (*F*_o_^2^ + 2*F*_c_^2^)/3
5554 reflections	(Δ/σ)_max_ = 0.002
330 parameters	Δρ_max_ = 0.50 e Å^−^^3^
1 restraint	Δρ_min_ = −0.37 e Å^−^^3^

### Special details

**Table:**

Geometry. All e.s.d.'s (except the e.s.d. in the dihedral angle between two l.s. planes) are estimated using the full covariance matrix. The cell e.s.d.'s are taken into account individually in the estimation of e.s.d.'s in distances, angles and torsion angles; correlations between e.s.d.'s in cell parameters are only used when they are defined by crystal symmetry. An approximate (isotropic) treatment of cell e.s.d.'s is used for estimating e.s.d.'s involving l.s. planes.
Refinement. Refinement of *F*^2^ against ALL reflections. The weighted *R*-factor *wR* and goodness of fit *S* are based on *F*^2^, conventional *R*-factors *R* are based on *F*, with *F* set to zero for negative *F*^2^. The threshold expression of *F*^2^ > σ(*F*^2^) is used only for calculating *R*-factors(gt) *etc*. and is not relevant to the choice of reflections for refinement. *R*-factors based on *F*^2^ are statistically about twice as large as those based on *F*, and *R*- factors based on ALL data will be even larger.

### Fractional atomic coordinates and isotropic or equivalent isotropic displacement parameters (Å^2^)

**Table:**

	*x*	*y*	*z*	*U*_iso_*/*U*_eq_	
N3	1.0640 (3)	1.0203 (2)	0.73941 (18)	0.0427 (4)	
Cd1	0.66437 (2)	0.504594 (16)	0.721726 (12)	0.03837 (6)	
O2	0.2288 (2)	0.84128 (17)	0.89531 (14)	0.0470 (4)	
O1	0.3826 (2)	0.68084 (17)	0.77635 (13)	0.0459 (4)	
C2	0.5023 (3)	0.66583 (19)	0.92189 (15)	0.0292 (4)	
N1	0.6429 (2)	0.55580 (16)	0.88029 (12)	0.0291 (3)	
C6	0.7749 (3)	0.4828 (2)	0.92965 (15)	0.0296 (4)	
O5	0.7653 (2)	0.69575 (17)	0.65361 (13)	0.0472 (4)	
C1	0.3595 (3)	0.7347 (2)	0.85908 (16)	0.0345 (4)	
O3	0.8962 (2)	0.31965 (15)	0.79612 (12)	0.0404 (3)	
O7	0.6154 (2)	0.31945 (16)	0.67431 (12)	0.0443 (4)	
N2	0.7307 (2)	0.50583 (17)	0.55184 (12)	0.0318 (3)	
O4	1.0562 (2)	0.29857 (19)	0.91131 (16)	0.0581 (5)	
O6	0.8533 (3)	0.80395 (19)	0.50805 (16)	0.0598 (5)	
C5	0.7702 (3)	0.5199 (2)	1.02547 (16)	0.0363 (4)	
H5	0.8626	0.4691	1.0595	0.044*	
C9	0.7864 (3)	0.6038 (2)	0.49661 (16)	0.0352 (4)	
C4	0.6252 (3)	0.6340 (2)	1.06974 (16)	0.0385 (5)	
H4	0.6193	0.6606	1.1342	0.046*	
C3	0.4892 (3)	0.7083 (2)	1.01798 (16)	0.0355 (4)	
H3	0.3909	0.7851	1.0469	0.043*	
C7	0.9229 (3)	0.3556 (2)	0.87480 (16)	0.0340 (4)	
C14	0.6464 (3)	0.3028 (2)	0.58002 (17)	0.0382 (5)	
C13	0.7083 (3)	0.4078 (2)	0.50696 (16)	0.0330 (4)	
C8	0.8043 (3)	0.7107 (2)	0.55696 (19)	0.0408 (5)	
O8	0.6343 (3)	0.20351 (18)	0.54186 (14)	0.0550 (5)	
N4	0.6575 (3)	0.9894 (2)	0.71014 (17)	0.0395 (4)	
C16	0.7838 (3)	0.9958 (2)	0.76668 (16)	0.0362 (4)	
H16	0.7338	1.0911	0.7957	0.043*	
C12	0.7429 (3)	0.4046 (3)	0.40160 (17)	0.0416 (5)	
H12	0.7277	0.3358	0.3706	0.050*	
C15	0.9497 (3)	0.9831 (2)	0.69164 (18)	0.0410 (5)	
H15A	1.0104	0.8845	0.6697	0.049*	
H15B	0.9212	1.0482	0.6317	0.049*	
C11	0.8002 (3)	0.5050 (3)	0.34338 (18)	0.0505 (6)	
H11	0.8236	0.5048	0.2724	0.061*	
C10	0.8232 (3)	0.6062 (3)	0.39052 (18)	0.0450 (5)	
H10	0.8623	0.6743	0.3520	0.054*	
C17	0.8098 (4)	0.8803 (3)	0.8543 (2)	0.0649 (8)	
H17A	0.7007	0.8971	0.8997	0.097*	
H17B	0.8906	0.8847	0.8910	0.097*	
H17C	0.8545	0.7861	0.8277	0.097*	
O9	0.3551 (3)	0.0640 (3)	0.8606 (2)	0.0659 (6)	
O10	0.3774 (3)	0.9659 (3)	0.60376 (17)	0.0623 (5)	
H4C	0.553 (4)	1.013 (3)	0.754 (2)	0.050 (8)*	
H4B	0.696 (4)	0.906 (3)	0.690 (2)	0.057 (8)*	
H4A	0.651 (4)	1.054 (3)	0.655 (2)	0.059 (8)*	
H9A	0.276 (6)	0.135 (5)	0.869 (3)	0.090 (13)*	
H10B	0.404 (5)	0.899 (4)	0.577 (3)	0.076 (13)*	
H10A	0.341 (8)	1.039 (7)	0.558 (5)	0.16 (2)*	
H9B	0.317 (5)	−0.001 (4)	0.883 (3)	0.081 (12)*	
H3C	1.100 (4)	0.957 (3)	0.7943 (18)	0.061 (9)*	
H3A	1.159 (4)	1.011 (3)	0.693 (2)	0.057 (8)*	
H3B	1.013 (4)	1.108 (4)	0.758 (2)	0.061 (9)*	

### Atomic displacement parameters (Å^2^)

**Table:**

	*U*^11^	*U*^22^	*U*^33^	*U*^12^	*U*^13^	*U*^23^
N3	0.0314 (10)	0.0350 (10)	0.0587 (13)	−0.0071 (8)	−0.0084 (10)	−0.0124 (9)
Cd1	0.05293 (11)	0.03011 (8)	0.02906 (8)	−0.01047 (6)	−0.01066 (6)	−0.00649 (5)
O2	0.0355 (9)	0.0356 (8)	0.0624 (11)	−0.0006 (7)	−0.0148 (8)	−0.0127 (7)
O1	0.0479 (9)	0.0413 (8)	0.0447 (9)	−0.0050 (7)	−0.0223 (7)	−0.0084 (7)
C2	0.0304 (10)	0.0243 (8)	0.0341 (9)	−0.0113 (7)	−0.0054 (8)	−0.0048 (7)
N1	0.0313 (9)	0.0251 (7)	0.0312 (8)	−0.0094 (6)	−0.0077 (7)	−0.0048 (6)
C6	0.0282 (10)	0.0279 (8)	0.0342 (9)	−0.0127 (7)	−0.0053 (8)	−0.0022 (7)
O5	0.0632 (11)	0.0349 (8)	0.0474 (9)	−0.0195 (7)	−0.0135 (8)	−0.0092 (7)
C1	0.0345 (11)	0.0270 (9)	0.0423 (11)	−0.0099 (8)	−0.0119 (9)	−0.0027 (8)
O3	0.0421 (9)	0.0311 (7)	0.0386 (8)	−0.0030 (6)	−0.0073 (7)	−0.0093 (6)
O7	0.0632 (11)	0.0335 (7)	0.0388 (8)	−0.0203 (7)	−0.0113 (8)	−0.0035 (6)
N2	0.0331 (9)	0.0270 (7)	0.0313 (8)	−0.0056 (7)	−0.0093 (7)	−0.0040 (6)
O4	0.0351 (9)	0.0504 (10)	0.0833 (14)	0.0012 (8)	−0.0240 (9)	−0.0222 (9)
O6	0.0699 (13)	0.0428 (9)	0.0738 (13)	−0.0306 (9)	−0.0176 (11)	0.0068 (9)
C5	0.0360 (11)	0.0402 (10)	0.0379 (11)	−0.0172 (9)	−0.0134 (9)	−0.0018 (8)
C9	0.0294 (10)	0.0316 (9)	0.0384 (10)	−0.0051 (8)	−0.0084 (8)	−0.0022 (8)
C4	0.0453 (13)	0.0426 (11)	0.0329 (10)	−0.0197 (10)	−0.0074 (9)	−0.0111 (8)
C3	0.0352 (11)	0.0335 (9)	0.0371 (10)	−0.0121 (8)	−0.0028 (9)	−0.0108 (8)
C7	0.0301 (10)	0.0257 (9)	0.0429 (11)	−0.0085 (8)	−0.0045 (9)	−0.0036 (8)
C14	0.0404 (12)	0.0270 (9)	0.0453 (12)	−0.0053 (8)	−0.0168 (10)	−0.0070 (8)
C13	0.0308 (10)	0.0286 (9)	0.0352 (10)	−0.0026 (8)	−0.0111 (8)	−0.0089 (7)
C8	0.0362 (12)	0.0285 (9)	0.0538 (13)	−0.0067 (8)	−0.0123 (10)	−0.0037 (9)
O8	0.0795 (13)	0.0384 (8)	0.0578 (10)	−0.0255 (9)	−0.0245 (10)	−0.0093 (7)
N4	0.0381 (11)	0.0341 (10)	0.0483 (11)	−0.0121 (8)	−0.0127 (9)	−0.0080 (8)
C16	0.0352 (11)	0.0325 (10)	0.0414 (11)	−0.0106 (8)	−0.0103 (9)	−0.0072 (8)
C12	0.0379 (12)	0.0452 (12)	0.0369 (11)	−0.0055 (9)	−0.0115 (9)	−0.0147 (9)
C15	0.0353 (12)	0.0378 (11)	0.0477 (12)	−0.0093 (9)	−0.0085 (10)	−0.0110 (9)
C11	0.0472 (14)	0.0666 (16)	0.0287 (10)	−0.0145 (12)	−0.0042 (10)	−0.0058 (10)
C10	0.0390 (12)	0.0520 (13)	0.0385 (11)	−0.0160 (10)	−0.0037 (10)	0.0018 (10)
C17	0.0664 (19)	0.0728 (19)	0.0647 (18)	−0.0363 (16)	−0.0286 (15)	0.0201 (15)
O9	0.0449 (11)	0.0434 (10)	0.0973 (17)	−0.0131 (9)	0.0117 (11)	−0.0213 (11)
O10	0.0782 (15)	0.0666 (14)	0.0503 (11)	−0.0389 (12)	−0.0044 (10)	−0.0085 (11)

### Geometric parameters (Å, °)

**Table:**

N3—C15	1.483 (3)	C9—C8	1.522 (3)
N3—H3C	0.924 (18)	C4—C3	1.381 (3)
N3—H3A	0.89 (3)	C4—H4	0.9300
N3—H3B	0.86 (3)	C3—H3	0.9300
Cd1—N1	2.2185 (16)	C14—O8	1.246 (3)
Cd1—N2	2.2236 (17)	C14—C13	1.517 (3)
Cd1—O7	2.2906 (16)	C13—C12	1.382 (3)
Cd1—O1	2.364 (2)	N4—C16	1.490 (3)
Cd1—O3	2.3965 (18)	N4—H4C	0.91 (3)
Cd1—O5	2.4197 (18)	N4—H4B	0.83 (3)
O2—C1	1.252 (3)	N4—H4A	0.91 (3)
O1—C1	1.251 (3)	C16—C17	1.515 (3)
C2—N1	1.333 (3)	C16—C15	1.518 (3)
C2—C3	1.388 (3)	C16—H16	0.9800
C2—C1	1.517 (3)	C12—C11	1.376 (4)
N1—C6	1.334 (3)	C12—H12	0.9300
C6—C5	1.383 (3)	C15—H15A	0.9700
C6—C7	1.522 (3)	C15—H15B	0.9700
O5—C8	1.268 (3)	C11—C10	1.384 (4)
O3—C7	1.266 (3)	C11—H11	0.9300
O7—C14	1.257 (3)	C10—H10	0.9300
N2—C9	1.334 (3)	C17—H17A	0.9600
N2—C13	1.335 (3)	C17—H17B	0.9600
O4—C7	1.231 (3)	C17—H17C	0.9600
O6—C8	1.234 (3)	O9—H9A	0.76 (4)
C5—C4	1.384 (3)	O9—H9B	0.84 (4)
C5—H5	0.9300	O10—H10B	0.74 (4)
C9—C10	1.387 (3)	O10—H10A	0.88 (6)
			
C15—N3—H3C	112.1 (19)	C4—C3—H3	120.7
C15—N3—H3A	107.1 (19)	C2—C3—H3	120.7
H3C—N3—H3A	107 (3)	O4—C7—O3	126.7 (2)
C15—N3—H3B	111 (2)	O4—C7—C6	116.74 (19)
H3C—N3—H3B	110 (3)	O3—C7—C6	116.52 (18)
H3A—N3—H3B	110 (3)	O8—C14—O7	124.7 (2)
N1—Cd1—N2	160.33 (6)	O8—C14—C13	117.2 (2)
N1—Cd1—O7	126.63 (6)	O7—C14—C13	118.04 (18)
N2—Cd1—O7	72.61 (6)	N2—C13—C12	120.8 (2)
N1—Cd1—O1	71.23 (7)	N2—C13—C14	114.95 (17)
N2—Cd1—O1	110.88 (7)	C12—C13—C14	124.22 (19)
O7—Cd1—O1	103.92 (7)	O6—C8—O5	126.1 (2)
N1—Cd1—O3	70.42 (6)	O6—C8—C9	117.6 (2)
N2—Cd1—O3	110.59 (7)	O5—C8—C9	116.27 (19)
O7—Cd1—O3	86.77 (6)	C16—N4—H4C	107.8 (18)
O1—Cd1—O3	138.50 (6)	C16—N4—H4B	108 (2)
N1—Cd1—O5	90.60 (6)	H4C—N4—H4B	113 (3)
N2—Cd1—O5	70.02 (6)	C16—N4—H4A	108.2 (19)
O7—Cd1—O5	142.62 (6)	H4C—N4—H4A	111 (3)
O1—Cd1—O5	90.16 (7)	H4B—N4—H4A	109 (3)
O3—Cd1—O5	105.44 (6)	N4—C16—C17	109.4 (2)
C1—O1—Cd1	115.64 (14)	N4—C16—C15	108.95 (18)
N1—C2—C3	120.81 (19)	C17—C16—C15	114.1 (2)
N1—C2—C1	115.44 (17)	N4—C16—H16	108.1
C3—C2—C1	123.71 (18)	C17—C16—H16	108.1
C2—N1—C6	121.19 (17)	C15—C16—H16	108.1
C2—N1—Cd1	118.94 (13)	C11—C12—C13	118.8 (2)
C6—N1—Cd1	119.73 (13)	C11—C12—H12	120.6
N1—C6—C5	120.86 (19)	C13—C12—H12	120.6
N1—C6—C7	115.26 (17)	N3—C15—C16	110.77 (19)
C5—C6—C7	123.83 (19)	N3—C15—H15A	109.5
C8—O5—Cd1	116.58 (14)	C16—C15—H15A	109.5
O1—C1—O2	126.0 (2)	N3—C15—H15B	109.5
O1—C1—C2	117.64 (18)	C16—C15—H15B	109.5
O2—C1—C2	116.35 (18)	H15A—C15—H15B	108.1
C7—O3—Cd1	114.43 (12)	C12—C11—C10	120.0 (2)
C14—O7—Cd1	116.65 (14)	C12—C11—H11	120.0
C9—N2—C13	121.17 (18)	C10—C11—H11	120.0
C9—N2—Cd1	121.10 (14)	C11—C10—C9	118.5 (2)
C13—N2—Cd1	117.70 (14)	C11—C10—H10	120.8
C6—C5—C4	118.7 (2)	C9—C10—H10	120.8
C6—C5—H5	120.6	C16—C17—H17A	109.5
C4—C5—H5	120.6	C16—C17—H17B	109.5
N2—C9—C10	120.7 (2)	H17A—C17—H17B	109.5
N2—C9—C8	116.00 (18)	C16—C17—H17C	109.5
C10—C9—C8	123.3 (2)	H17A—C17—H17C	109.5
C3—C4—C5	119.84 (19)	H17B—C17—H17C	109.5
C3—C4—H4	120.1	H9A—O9—H9B	104 (4)
C5—C4—H4	120.1	H10B—O10—H10A	106 (4)
C4—C3—C2	118.59 (19)		
			
N1—Cd1—O1—C1	9.37 (15)	O3—Cd1—N2—C9	−100.88 (15)
N2—Cd1—O1—C1	−149.82 (15)	O5—Cd1—N2—C9	−1.33 (15)
O7—Cd1—O1—C1	133.77 (16)	N1—Cd1—N2—C13	170.20 (16)
O3—Cd1—O1—C1	32.7 (2)	O7—Cd1—N2—C13	1.35 (14)
O5—Cd1—O1—C1	−81.21 (16)	O1—Cd1—N2—C13	−97.29 (15)
C3—C2—N1—C6	0.3 (3)	O3—Cd1—N2—C13	80.95 (15)
C1—C2—N1—C6	−177.55 (17)	O5—Cd1—N2—C13	−179.50 (16)
C3—C2—N1—Cd1	−175.32 (14)	N1—C6—C5—C4	0.3 (3)
C1—C2—N1—Cd1	6.9 (2)	C7—C6—C5—C4	−177.21 (19)
N2—Cd1—N1—C2	91.3 (2)	C13—N2—C9—C10	−0.2 (3)
O7—Cd1—N1—C2	−101.95 (15)	Cd1—N2—C9—C10	−178.30 (16)
O1—Cd1—N1—C2	−8.29 (14)	C13—N2—C9—C8	179.24 (18)
O3—Cd1—N1—C2	−172.14 (16)	Cd1—N2—C9—C8	1.1 (2)
O5—Cd1—N1—C2	81.67 (15)	C6—C5—C4—C3	−0.1 (3)
N2—Cd1—N1—C6	−84.3 (2)	C5—C4—C3—C2	−0.1 (3)
O7—Cd1—N1—C6	82.38 (16)	N1—C2—C3—C4	0.0 (3)
O1—Cd1—N1—C6	176.04 (16)	C1—C2—C3—C4	177.62 (19)
O3—Cd1—N1—C6	12.20 (13)	Cd1—O3—C7—O4	−160.3 (2)
O5—Cd1—N1—C6	−93.99 (15)	Cd1—O3—C7—C6	19.5 (2)
C2—N1—C6—C5	−0.4 (3)	N1—C6—C7—O4	170.5 (2)
Cd1—N1—C6—C5	175.12 (15)	C5—C6—C7—O4	−11.9 (3)
C2—N1—C6—C7	177.30 (17)	N1—C6—C7—O3	−9.4 (3)
Cd1—N1—C6—C7	−7.1 (2)	C5—C6—C7—O3	168.28 (19)
N1—Cd1—O5—C8	178.02 (17)	Cd1—O7—C14—O8	176.57 (18)
N2—Cd1—O5—C8	1.47 (16)	Cd1—O7—C14—C13	−1.5 (3)
O7—Cd1—O5—C8	2.8 (2)	C9—N2—C13—C12	0.2 (3)
O1—Cd1—O5—C8	−110.75 (17)	Cd1—N2—C13—C12	178.42 (15)
O3—Cd1—O5—C8	108.19 (17)	C9—N2—C13—C14	179.35 (18)
Cd1—O1—C1—O2	170.55 (18)	Cd1—N2—C13—C14	−2.5 (2)
Cd1—O1—C1—C2	−9.2 (2)	O8—C14—C13—N2	−175.54 (19)
N1—C2—C1—O1	2.1 (3)	O7—C14—C13—N2	2.6 (3)
C3—C2—C1—O1	−175.7 (2)	O8—C14—C13—C12	3.5 (3)
N1—C2—C1—O2	−177.69 (19)	O7—C14—C13—C12	−178.3 (2)
C3—C2—C1—O2	4.6 (3)	Cd1—O5—C8—O6	177.91 (19)
N1—Cd1—O3—C7	−17.16 (14)	Cd1—O5—C8—C9	−1.4 (2)
N2—Cd1—O3—C7	141.91 (15)	N2—C9—C8—O6	−179.1 (2)
O7—Cd1—O3—C7	−148.03 (15)	C10—C9—C8—O6	0.3 (3)
O1—Cd1—O3—C7	−40.58 (19)	N2—C9—C8—O5	0.3 (3)
O5—Cd1—O3—C7	67.86 (15)	C10—C9—C8—O5	179.7 (2)
N1—Cd1—O7—C14	−175.19 (15)	N2—C13—C12—C11	−0.3 (3)
N2—Cd1—O7—C14	0.15 (15)	C14—C13—C12—C11	−179.3 (2)
O1—Cd1—O7—C14	108.04 (17)	N4—C16—C15—N3	167.86 (18)
O3—Cd1—O7—C14	−112.60 (17)	C17—C16—C15—N3	−69.6 (3)
O5—Cd1—O7—C14	−1.2 (2)	C13—C12—C11—C10	0.3 (4)
N1—Cd1—N2—C9	−11.6 (3)	C12—C11—C10—C9	−0.3 (4)
O7—Cd1—N2—C9	179.53 (17)	N2—C9—C10—C11	0.2 (3)
O1—Cd1—N2—C9	80.88 (16)	C8—C9—C10—C11	−179.2 (2)

### Hydrogen-bond geometry (Å, °)

**Table:**

*D*—H···*A*	*D*—H	H···*A*	*D*···*A*	*D*—H···*A*
N3—H3A···O10^i^	0.89 (3)	1.90 (3)	2.780 (3)	172 (3)
N3—H3B···O3^ii^	0.86 (3)	2.04 (3)	2.899 (3)	177 (3)
N3—H3C···O2^i^	0.92 (2)	1.89 (2)	2.790 (3)	164 (3)
N4—H4A···O8^ii^	0.91 (3)	1.96 (3)	2.870 (3)	176 (3)
N4—H4B···O5	0.83 (3)	2.06 (3)	2.889 (3)	172 (3)
N4—H4C···O9^ii^	0.91 (3)	1.90 (3)	2.803 (3)	170 (3)
O9—H9A···O4^iii^	0.76 (4)	1.96 (4)	2.708 (3)	170 (4)
O9—H9B···O2^iv^	0.84 (4)	2.00 (4)	2.827 (3)	165 (4)
O10—H10A···O6^v^	0.88 (6)	2.04 (6)	2.848 (4)	151 (6)
O10—H10B···O8^vi^	0.74 (4)	2.19 (4)	2.835 (3)	147 (4)
C10—H10···O3^vii^	0.93	2.54	3.298 (3)	139
C12—H12···O1^vi^	0.93	2.44	3.200 (3)	139

Symmetry codes: (i) *x*+1, *y*, *z*; (ii) *x*, *y*+1, *z*; (iii) *x*−1, *y*, *z*; (iv) *x*, *y*−1, *z*; (v) −*x*+1, −*y*+2, −*z*+1; (vi) −*x*+1, −*y*+1, −*z*+1; (vii) −*x*+2, −*y*+1, −*z*+1.
